# Pheniramine Maleate is more effective than Lidocaine on Fentanyl Induced Cough

**DOI:** 10.12669/pjms.323.9496

**Published:** 2016

**Authors:** Ozgur Ozmen, Duygu Kara, Emine Uzlas Karaman, Fatma Karakoc, Muhammet Ahmet Karakaya, Zakir Arslan

**Affiliations:** 1 Dr. Ozgur Ozmen, Department of Anesthesiology and Reanimation, Erzurum Regional Training and Research Hospital, Erzurum, Turkey; 2 Dr. Duygu Kara, Department of Anesthesiology and Reanimation, Erzurum Regional Training and Research Hospital, Erzurum, Turkey; 3 Dr. Emine Uzlas Karaman, Department of Anesthesiology and Reanimation, Erzurum Regional Training and Research Hospital, Erzurum, Turkey; 4 Dr. Fatma Karakoc, Department of Anesthesiology and Reanimation, Erzurum Regional Training and Research Hospital, Erzurum, Turkey; 5 Dr. Muhammet Ahmet Karakaya, Department of Anesthesiology and Reanimation, Erzurum Regional Training and Research Hospital, Erzurum, Turkey; 6 Dr. Zakir Arslan, Department of Anesthesiology and Reanimation, Erzurum Regional Training and Research Hospital, Erzurum, Turkey

**Keywords:** Fentanyl, Pheniramine Maleate, Lidocaine, Cough

## Abstract

**Objective:**

Fentanyl is frequently used during anesthesia induction. The use of fentanyl can cause cough through different mechanisms. Here, we aimed to investigate effects of pheniramine maleate (PM), an antihistaminic agent, and compare it with lidocaine on fentanyl induced cough.

**Methods:**

This is a randomized double-blind prospective clinical study of ASA I-II, 120 patients scheduled for elective abdominal surgery. Patients were administered drugs intravenously and randomly allocated into three groups: Group C (2 ml 0.9 % normal saline), Group L (1mg/kg lidocaine), and Group F (PM 45.5 mg). 90 seconds after administration, 2µ/kg fentanyl was applied in three seconds to all patients. Severity of cough (mild: 1-2, moderate: 3-5, severe> 5), time of the cough and vital parameters were recorded 90 seconds after fentanyl injection.

**Results:**

Eight patients (25%) in Group C had fentanyl induced cough whereas three patients (7.5%) in Group L and one patient (2.5%) in Group F experienced this phenomenon. There was statistically significant difference between Group F and Group C (p<0.05); however, differences between Group L and Group C or Group F and Group L were not statistically significant (p>0.05).

**Conclusions:**

Pheniramine Maleate 45.5 mg is better that placebo and as effective as lidocaine to prevent fentanyl induced cough.

## INTRODUCTION

Fentanyl is a frequently used synthetic opioid agent in anesthesia induction due to its high analgesic efficacy, fast onset of effect, reducing effect on cardiovascular response.^1^,^2^ Despite opioid agonists have antitussive effects; fentanyl, is an an opioid which can cause cough if administered as bolus intravenously during anesthesia induction.^3^,^4^ Cough during general anesthesia induction can cause several adverse effects requiring immediate action because of increased intracranial, intraocular and intra-abdominal pressures.^5^,^6^

Supraglottic obstruction by soft tissue, fentanyl induced histamine release and/or sudden adduction in vocal cord muscles due to opioid induced muscle rigidity may be the mechanism of opioid induced cough.^7^,^8^

In addition, smoking leads to respiratory symptoms, such as cough, increased mucus secretion, and airway inflammation. In our routine anesthesia management, the incidence of fentanyl-induced cough does not seem to be more frequent in current smokers, contradicting expectations regarding the impact of smoking on cough. Studies on fentanyl-induced cough have generally excluded smokers from analysis and the effects of smoking was evaluated in a few studies. There are many studies regarding incidence of fentanyl induced cough and have different results. Cough incidence varies with fentanyl dose.^3^,^9^ Yakici et al. reported injection velocity of fentanyl also effects cough incidence^10^, however Akcaboy et al. found no effect of injection velocity on cough incidence.^11^ Several methods are tried to prevent fentanyl induced cough.^3^,^4^,^11^-^13^ But, in literature there is no data about the effect of pheniramine maleat on fentanyl induced cough.

Our objective in this study was to determine effect of pheniramine maleate with smoking history on the occurrence of fentanyl-induced cough. based on references suggesting histamine release as one of its possible causes and to compare effects of pheniramine maleate and lidocaine, which is commonly used in this indication, on prevention of fentanyl induced cough.

## METHODS

The study was conducted in our research hospital between May 2015 and August 2015 and designed as a randomized, double-blinded and three-way study. After obtaining Erzurum Regional Training and Research Hospital Ethical Committee approval (number 2015/9-62) and written informed consents from patients; 120 ASA physical status I-II patients between ages of 18 and 65 years who were scheduled for elective abdominal surgery and general anesthesia were included. Patients were allocated into three groups; control group (Group C), pheniramine maleate group (Group F) and lidocaine group (Group L) having 40 patients each; randomized by using sealed and numbered envelopes. An anesthesiologist was responsible for preparation of study drugs and allocation process. Observers and patients were unaware of the groups for study.

Patients between ages of 18 and 65 years, ASA (American Society of Anesthesiologists) physical status I-II, who were scheduled for elective open abdominal surgery, smokers and non-smokers, had not received premedication as it may effect the cough incidence and did not have allergies to local anesthetics and any other drugs were included in this study.

Patients who received premedication; had throat infection less than three weeks before surgery, COPD (Chronic Obstructive Pulmonary Disease), chronic cough, history of drug allergy or asthma and patients who were using angiotensin converting enzyme inhibitors, bronchodilators and/or steroids for the last month were excluded from the study.

Our power analysis showed that using a sample size of 40 patients per group achieves 87 % power to detect an effect size (W) of 0.5 using 2 degrees of freedom Chi-Square Test with a significance level (alpha) of 0.05.

The groups having 40 patients each; randomized by using sealed and numbered envelopes and were admitted to operating room without any premedication. Routine monitoring (pulse oximetry, non-invasive blood pressure, ECG) was implemented and vital signs were recorded. Intravenous line was applied with 22G cannula on dorsal side of the hand and 0.9% NaCl infusion was started at the rate of 3ml/kg/hour.

For anesthesia induction, 1 ampoule of pheniramine maleate 45.5 mg iv bolus (Avil, 2 ml ampoule, Sandoz, Turkey) was administered to patients in Group F, 1mg/kg lidocaine (Aritmal 2%, 5 ml, Osel drug, Turkey) to Group L and 2 ml of 0.9% NaCl to Group C intravenously and all drugs were diluted with 5 ml of saline solution. After 90 seconds, 2µg/kg fentanyl (Fentanyl 0.05 mg/ml, 10 ampoule, Johnson&Johnson, Belgium) was administered intravenously in 3 seconds. Ninety seconds after fentanyl injection, based on the number of coughs observed, cough severity was graded as mild (1–2), moderate (3–5), or severe (>5) and vital parameters were recorded by an independent observer. Fentanyl related side effects were monitored and recorded. Anesthesia induction was completed with 2.5 mg/kg propofol and 0.6 mg/kg rocuronium. Patients were ventilated with face mask for 2.5 minutes and intubated orotracheally. Mechanic ventilation was implemented with 7 ml/kg tidal volume and respiratory rate of 12 per minute.

The distribution of the variables was evaluated for normality using the Kolmogorov-Smirnov and histogram tests. Descriptive statistics was expressed as the means±standard deviation (SD). Categorical variables were analyzed using the chi-square test. The normally distributed data comprising continuous variables were analyzed using One Way ANOVA. Otherwise, the Kruskal Wallis test was used. A value of P< 0.05 was considered statistically significant. IBM SPSS 20.0 (SPSS Inc., Chicago, Illinois, USA) software program was used to perform the statistical analysis.

## RESULTS

There was no significant difference between groups regarding age, gender, weight and ASA distributions (p>0.05). Demographic data were shown in Table-I.

**Table-I:** Patient characteristics in three groups.

	*Group C n=40*	*Group F n=40*	*Group L n=40*	p*
Age (years)	39.90±11.56	43.12±11.29	41.30±11.00	0.406^a^
Male (F/M)	21/19	19/21	22/18	0.792^b^
Weight (kg)	72.52±10.90	76.90±11.36	74.95±9.2	0.281^a^
Height (cm)	167.65±8.8	168.17±8.69	167.80±9.14	0.976^a^
ASA (I/II)	9/31	8/32	7/33	0.855^b^

Values are given as number or mean±SD, Group C, Control Group; Group F, Feniramin; Group L, Lidokain.

* P>0.05 between the cases.

a Kruskal-Wallis test

b Chi-square test.

Number of patients who had fentanyl induced cough, distribution among groups and severity of the cough are shown in Table-II. There was a statistically significant difference between groups regarding cough frequency (p=0.027). One patient in Group F (2.5%), 8 in Group C (25%) and 3 in Group L (7.5%) had fentanyl induced cough. Cough frequency was lower in Group F compared to Group C (p=0.029). There was no statistical difference between Group L and Group C or Group F and Group L (p=0.193 and p=0.615 respectively) by means of cough frequency. Only one out of 12 patients had severe, 4 had moderate and others had mild cough.

**Table-II:** The comparison of cough characteristics in three groups.

	*Group C n=40*	*Group F n=40*	*Group L n=40*	*p*
Cough incidence, n (%)	8 (20%)	1 (2.5%)	3(7.5%)	0.027*
Mild cough (1-2 times), n (%)	4 (50%)	1(100%)	2 (67%)	NS
Moderate cough (3-5 times), n (%)	3 (37.5%)	0	1 (33%)	
Severe cough (> 5 times), n (%)	1 (12.5%)	0	0	

Values are given as number or percentage

* Chi-square test, NS: non-significant.

Data for time of fentanyl induced cough are shown in Table-III. After fentanyl injection, 6 patients had cough in first 10 seconds, 6 occurred between 10 and 15 seconds; none of the patients had cough after 15 seconds.

**Table-III:** The time interval between fentanyl injection and onset of cough symptom.

	*Group C n=8*	*Group F n=1*	*Group L n=3*
1-10 second n (%)	5 (62.5%)	0	1 (33.3%)
11-15 second n (%)	3 (37.5%)	1 (100%)	2 (66.7%)

Number of patients who had tobacco consumption and cough data of these patients are shown in Table-IV. There was no statistically significant difference between groups regarding number of patients who had tobacco consumption (p=0.214). Nineteen patients were smokers in Group F but none of them had cough. Twelve patients in Group C and 13 patients in Group L had tobacco consumption. Of the smokers in Group C and Group L, only one patient in each group had cough.

**Table-IV:** Comparison of cough characteristics of patients with tobacco use.

	*Group C n=40*	*Group F n=40*	*Group L n=40*	*p*
Smoker n (%)	12 (30 %)	19 (47.5%)	13(32.5 %)	0.214*

Number of patients (n)	1	0	1	

Values are given as number or percentage,

* Chi-square test.

## DISCUSSION

Cough during general anesthesia induction can cause several conditions requiring immediate action because of increased intracranial, intraocular and intraabdominal pressures.^5^,^6^ Fentanyl use can cause histamine release and bronchospasm especially in children with allergy history; this may in return can cause difficulty in ventilation.^14^

In our study, we observed that 2µg/kg fentanyl given in 3 seconds causes cough in 20% of the patients. The use of pheniramine maleate before induction reduces this rate to 2.5% and lidocaine reduces it to 7.5%. Fentanyl induced cough occurred in the first 15 seconds. There was no significant relation between smoking and cough occurrence.

Opioid related cough is a common phenomenon during intravenous anesthesia induction.^11^-^13^ Cause of opioid induced cough may be sudden adduction in vocal cord muscles caused by opioid induced muscle rigidity, supraglottic obstruction by soft tissues; or histamine release from mast cells in respiratory tract.^7^,^8^ Agarwal reported reduced frequency of fentanyl induced cough in patients who use bronchodilator agents.^15^ This suggests that broncho constructive or allergic mediators such as histamine may be the cause of fentanyl induced cough.

Frequency of fentanyl induced cough has a wide range and it varies with injection site, velocity, dosage and concentration of fentanyl.^11^,^16^ Different studies show 21.3% to 28% cough rates with 2 µg kg^-1^ fentanyl.^9^,^15^,^17^,^18^ Lin et al. reported 65% cough rate with 2.5 µg kg^-1^ fentanyl.^13^ Pandley et al. reported 34.2% and 35% in two different studies with 3 µg kg^-1^ fentanyl.^4^,^19^ Lui et al. reported 43% frequency with 5 µg kg^-1^ fentanyl.^20^ We used 2 µg kg^-1^ fentanyl and found that fentanyl induced cough frequency was 20%.

Studies about fentanyl induced cough also examined effect of velocity of injection on cough frequency and reported different results. Yakici et al. compared 5 seconds and 30 seconds and reported 23% and 3.5% cough frequencies, respectively.^10^ Akcaboy et al. compared 2 seconds and 20 seconds and reported 11.4% and 8.6% cough frequencies, respectively.^11^ Schäpermeier et al. compared 2 seconds, 5 seconds and 10 seconds and reported 2%, 3% and 6% cough frequencies, respectively.^21^ We standardized the velocity of injection to 3 seconds in our study.

Studies aiming to prevent fentanyl induced cough used many agents including propofol, atropine, ketamine, morphine, midazolam, terbutaline, salbutamol, lidocaine and ephedrine.^4^,^12^-^15^,^19^,^20^,^22^,^23^ Lui et al. reduced cough frequency from 43% to 3% by using terbutaline via inhalation.^20^ Agarwal et al. reduced frequency from 28% to 6% by using salbutamol via inhalation.^15^ Lin et al. reduced frequency from 65% to 14% with 2mg/kg intravenous lidocaine, to 37% with 0.6 mg/kg propofol and to 21% with 5 mg ephedrine.^13^ Tang et al. used propofol in doses of 1 mg/kg, 1.5 mg/kg and 2 mg/kg and reduced frequency from 80% to 40%, 6.7% and 3.3% respectively.^12^ Pandey et al. examined 3 µg kg^-1^ intravenous fentanyl in two different studies and reported 35% cough frequency; they used 0.5 mg/kg, 1 mg/kg and 1 mg/kg intravenous lidocaine and reported 13-15% cough frequency.^4^,^19^ We observed a decrease in cough frequency from 20% to 7.5% by using 1 mg/kg lidocaine, however this difference between lidocaine group and control group was not statistically significant.

Neuromuscular blocker agents and opioids which are used for anesthesia induction both cause histamine release.^24^,^25^ Histamine release also seems to play a role in mechanism of fentanyl induced cough.^8^ Taking these studies into consideration, we used pheniramine maleate which is an antihistamine and also has sedative properties during anesthesia induction to prevent fentanyl induced cough. Cough frequency in pheniramine maleate group was 2.5%. The difference in cough frequencies between pheniramine maleate group and control group was statistically significant (p<0.05).

Akcaboy et al. defined fentanyl induced cough as usually benign.^11^ We also observed that only 1 in 12 patient had severe cough. Four patients had moderate, 7 patients had mild cough. Our results also suggest that fentanyl induced cough has a benign character.

Studies show that fentanyl induced cough occurs within less than 30 seconds after injection.^10^,^16^,^26^ In our study time of the cough never passed 15 seconds in any of the groups. We observed that 50% of our patients had coughed in first 10 seconds and 50% coughed between 10^th^ and 15^th^ seconds.

In this study, smoking rate among our 120 patients was 37%. Only 0.88% of these patients had cough. Largest number of smokers was in Group F but none of these patients had cough.

In conclusion, we found that pheniramine maleate which is an antihistaminic is more effective than lidocain and can be considered as an alternative method to prevent fentanyl induced cough during anesthesia induction.
